# *Bacillus subtilis *SbcC protein plays an important role in DNA inter-strand cross-link repair

**DOI:** 10.1186/1471-2199-7-20

**Published:** 2006-06-16

**Authors:** Judita Mascarenhas, Humberto Sanchez, Serkalem Tadesse, Dawit Kidane, Mahalakshmi Krisnamurthy, Juan C Alonso, Peter L Graumann

**Affiliations:** 1 Institut für Mikrobiologie, Albert-Ludwigs Universität Freiburg, Stefan Meier Str. 19, 79104 Freiburg, Germany; 2 Departamento de Biotecnología Microbiana, Centro Nacional de Biotecnología, CSIC, C/Darwin 3, Campus Universidad Autónoma de Madrid, Cantoblanco, 28049 Madrid, Spain; 3 Institut für Klinische Zytobiologie und Zytopathologie, Robert-Koch-Straße 6, 35037 Marburg, Germany

## Abstract

**Background:**

Several distinct pathways for the repair of damaged DNA exist in all cells. DNA modifications are repaired by base excision or nucleotide excision repair, while DNA double strand breaks (DSBs) can be repaired through direct joining of broken ends (non homologous end joining, NHEJ) or through recombination with the non broken sister chromosome (homologous recombination, HR). Rad50 protein plays an important role in repair of DNA damage in eukaryotic cells, and forms a complex with the Mre11 nuclease. The prokaryotic ortholog of Rad50, SbcC, also forms a complex with a nuclease, SbcD, in *Escherichia coli*, and has been implicated in the removal of hairpin structures that can arise during DNA replication. Ku protein is a component of the NHEJ pathway in pro- and eukaryotic cells.

**Results:**

A deletion of the *sbcC *gene rendered *Bacillus subtilis *cells sensitive to DNA damage caused by Mitomycin C (MMC) or by gamma irradiation. The deletion of the *sbcC *gene in a *recN *mutant background increased the sensitivity of the single *recN *mutant strain. SbcC was also non-epistatic with AddAB (analog of *Escherichia coli *RecBCD), but epistatic with RecA. A deletion of the *ykoV *gene encoding the *B. subtilis *Ku protein in a *sbcC *mutant strain did not resulted in an increase in sensitivity towards MMC and gamma irradiation, but exacerbated the phenotype of a *recN *or a *recA *mutant strain. In exponentially growing cells, SbcC-GFP was present throughout the cells, or as a central focus in rare cases. Upon induction of DNA damage, SbcC formed 1, rarely 2, foci on the nucleoids. Different to RecN protein, which forms repair centers at any location on the nucleoids, SbcC foci mostly co-localized with the DNA polymerase complex. In contrast to this, AddA-GFP or AddB-GFP did not form detectable foci upon addition of MMC.

**Conclusion:**

Our experiments show that SbcC plays an important role in the repair of DNA inter-strand cross-links (induced by MMC), most likely through HR, and suggest that NHEJ via Ku serves as a backup DNA repair system. The cell biological experiments show that SbcC functions in close proximity to the replication machinery, suggesting that SbcC may act on stalled or collapsed replication forks. Our results show that different patterns of localization exist for DNA repair proteins, and that the *B. subtilis *SMC proteins RecN and SbcC play distinct roles in the repair of DNA damage.

## Background

All organisms need to ensure the integrity of their genome. A major threat are inter-strand cross-links and double strand breaks (DSBs) in the DNA that can cause cell death or cellular transformation. The repair of inter-strand cross-links appears to be achieved via two pathways, including proteins involved in nucleotide excision repair, and DNA polymerase II or proteins mediating homologous recombination (HR) [1]. At least two pathways also exist for the repair of DSBs. These can be repaired through direct end joining (non homologous end joining, NHEJ), but this process creates the danger of connecting the wrong ends, and of losing genetic information. In eukaryotic and in some prokaryotic cells, Ku protein binds to broken DNA ends, protects them from exonucleolytic degradation and specifically recruits a dedicated DNA ligase that seals the ends [2,3]. *In Bacillus subtilis*, deletion of the genes encoding for Ku and DNA ligase (*ykoV *and *ykoU*) leads to sensitivity to DSBs during stationary phase [4], showing that this pathway has an important function in non-growing cells. A much less error prone pathway is DNA repair through HR, in which a variety of proteins uses the intact sister chromosome to fix a DSB [5-7]. Central to this pathway is strand exchange protein RecA (Rad51 in eukaryotes) that promotes the annealing of the 3'-single-stranded (ss) DNA from the broken chromosome with the homologous sister copy and thereby catalyzes HR. In *B. subtilis*, the AddAB (nuclease/helicase) complex (RecBCD in *Escherichia coli*) or alternatively, RecQ/RecS/RecJ act upstream of RecA. These enzymes generate a ssDNA region at the DSB, which is used for strand exchange [8]. AddAB or RecO/RecR load or help to load RecA onto the 3' ssDNA end [9]. In eukaryotes, the Rad50/Mre11/Xrs2 (Nbs1 in human cells) complex is thought to perform the degradation of one DNA strand to generate a 3' ssDNA overhang. Downstream of RecA, different DNA helicases, such as RecG, and the RuvABC complex are involved in the formation of Holliday junctions and in the resolution of crossovers [6], while DNA polymerase I is required to fill ssDNA gaps.

The eukaryotic Rad50 complex plays an important role in DNA repair in eukaryotes through HR [10,11], and is apparently also involved in NHEJ through bridging of the broken DNA ends [12,13]. Mre11 has endo- and exonuclease activity in the presence of Rad50 [14]. Rad50 has been shown to bind to DNA in an ATP-dependent manner [15], although the Rad50 complex was found to be able to bind to DNA in the absence of ATP [16]; DNA end-bridging is ATP independent [17]. Rad50 belongs to the family of SMC proteins that share a similar modular structure. These proteins are composed of conserved N- and C- terminal domains that are separated by two long coiled coil domains and a central hinge domain. Two monomers invariably form a dimer through specific interaction of the hinge domains, creating a symmetrical dimer with a central hinge, two long coiled coil arms and two ATPase head domains [18,19]. In Rad50, the coiled coil arms lack a hinge domain. Instead, the coiled coils can connect in a dynamic manner via a Zn bridge formed by an invariable CxxC motif, which may mediate end bridging [20]. SMC proteins are central components of several essential protein complexes in pro- and eukaryotes, and mediate a wide range of chromosome dynamics [21]. In bacteria, the SMC complex localizes as two foci, one within each cell half, and actively compacts and organises chromosomes from these sites [22]. The chromosome is replicated in and moves through the centrally located DNA polymerase complex [23]. Thus, bacterial chromosomes are segregated during ongoing replication, while both processes occur at different times during the cell cycle in eukaryotic cells.

In *E. coli*, the Rad50 ortholog, SbcC, forms a complex with the Mre11-homologous SbcD nuclease [24], which has been shown to be involved in cleavage of DNA hairpins that can occur during replication [25]. Many other bacteria possess *sbcC *homologous genes, but no reports on SbcC in other bacteria are available, and it is unclear if SbcC plays a similar role in DNA repair as Rad50 in eukaryotes.

In recent years, repair of DNA DSBs has been visualized in live eukaryotic cells, giving vital insight into the function of proteins. The Rad50 complex, Rad51, Rad52 and other repair proteins have been shown to form discrete foci on damaged chromosomes, and to dynamically interact within the repair structures, or to form distinct repair foci [26-30]. In bacteria, RecA, RecN, RecO and RecF have been shown to co-localize to discrete centres on the nucleoids after induction of DNA damage, showing that bacteria also form DNA repair centres after DNA damage [31,32]. RecN is also a member of the SMC protein family, plays an important function in DNA repair via HR, and has ssDNA-binding activity *in vitro *[8,33].

In this work, we have investigated the role of prokaryotic SbcC in DNA repair, and show it has an important function in this process, which is different from that of RecN. We have visualized the dynamic formation of defined SbcC foci on the DNA in response to DNA damage. The foci were generally coincident with the position of the replication machinery, and therefore most likely represent stalled replication forks, whose restart may be facilitated by the action of SbcC.

## Results

### *B. subtilis *SbcC is involved in DNA repair

**Figure 1:**
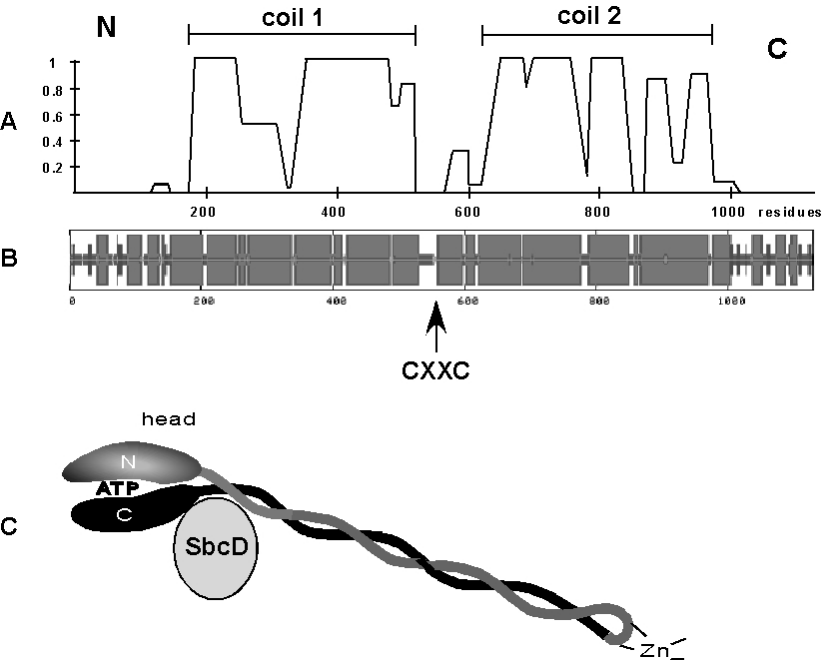
A) Analysis of the probability of coiled coil regions within *B. subtilis *SbcC, using programs from . The position of the corresponding CXXC motif forming a Zn bridge in Rad50 [20] is indicated. B) Analysis of the formation of α-helices in SbcC. C) Putative structure of SbcC, and putative binding of SbcD, according to data obtained for Rad50 protein [18, 56]. SbcC has the potential to form a dimer with another SbcC monomer via the Zn-bridge. It is interesting to note that in spite of their similarity, the SbcCD and Rad50/Mre11 complexes have a different arrangement *in vitro *[57].

**Figure 2:**
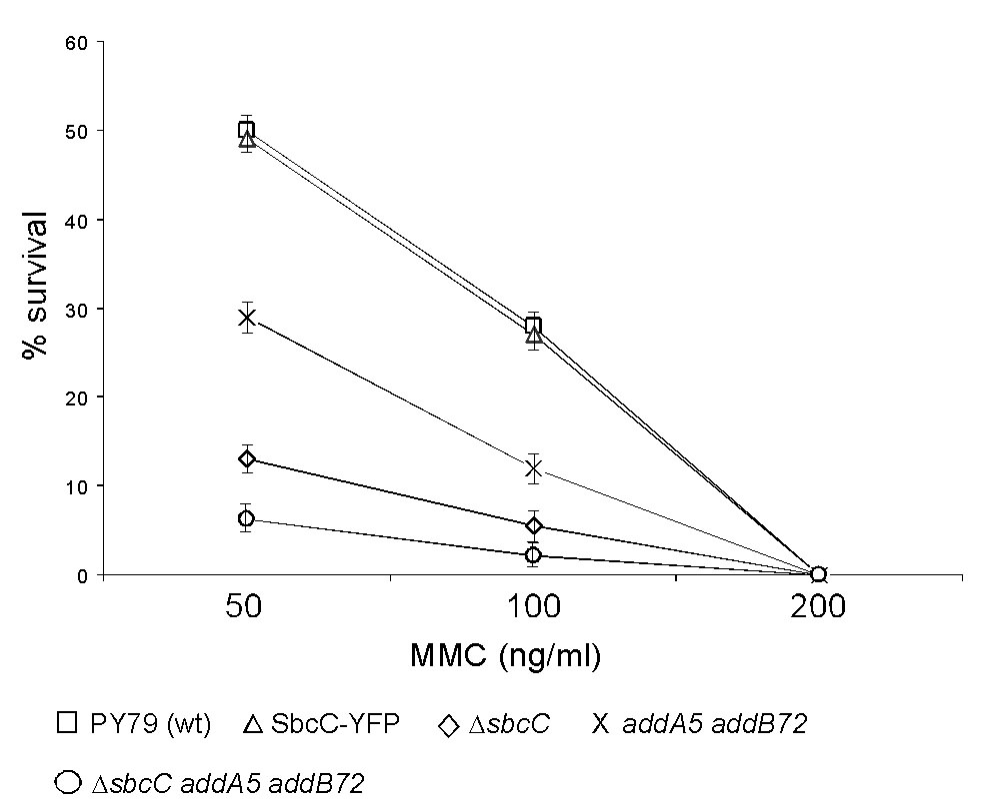
Survival of *Bacillus subtilis *wild type or of mutant cells after addition of different doses of MMC for 20 min. The strains used are indicated by the relevant genotype. At least three independent experiments were performed, bars denote standard deviations.

**Figure 3:**
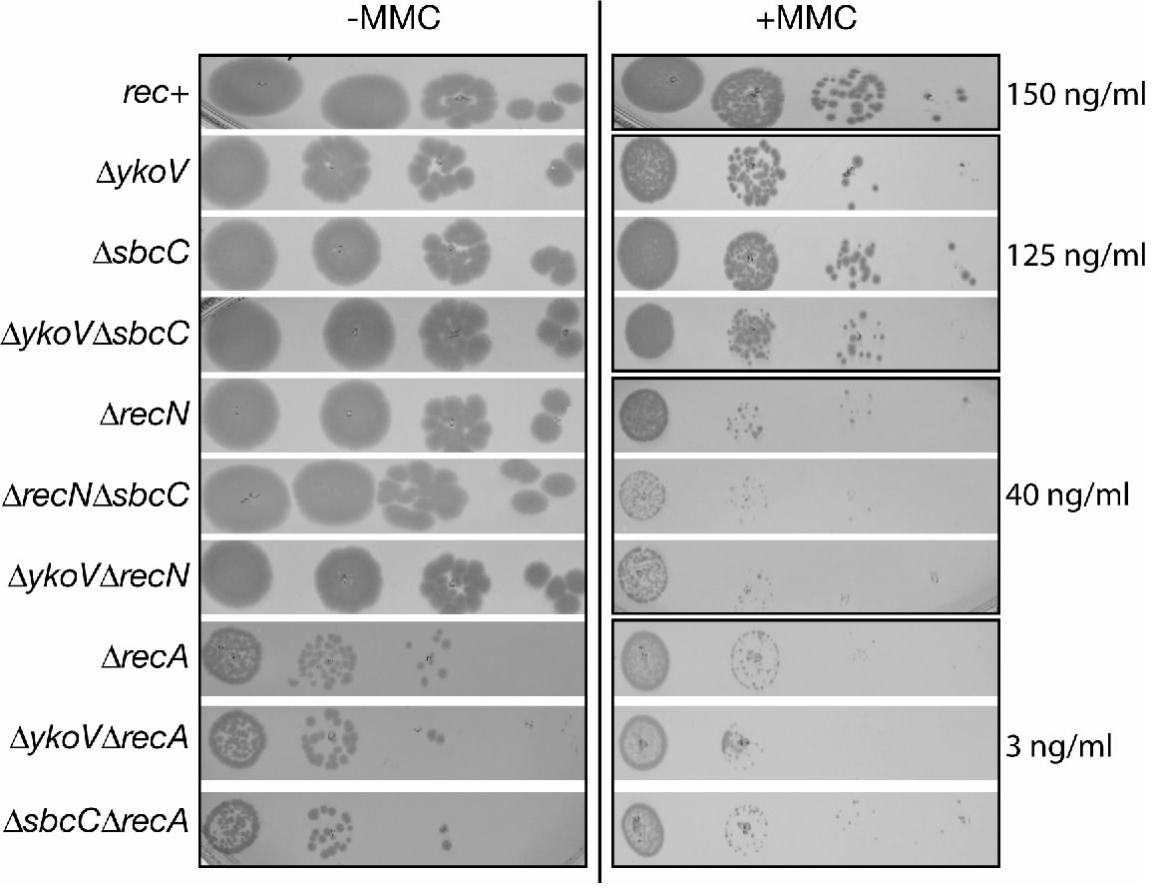
DNA repair sensitivity of exponential-phase Δ*sbcC *and Δ*ykoV *mutants to the killing action of MMC. The strains used are indicated by the relevant genotype. Serial dilutions of a culture of each strain were plated in selective medium containing the indicated concentration of MMC. Wild type cells were exposed to 150 ng/ml, Δ*sbcC*, Δ*ykoV *or Δ*sbcC *Δ*ykoV *to 125 ng/ml, Δ*recN*, Δ*sbcC *Δ*recN *or Δ*ykoV *Δ*recN *to 40 ng/ml and Δ*recA*, Δ*ykoV *Δ*recA *or Δ*sbcC *Δ*recA *were exposed to 3 ng/ml of MMC. At least three independent experiments were performed and one of them is presented.

**Figure 4:**
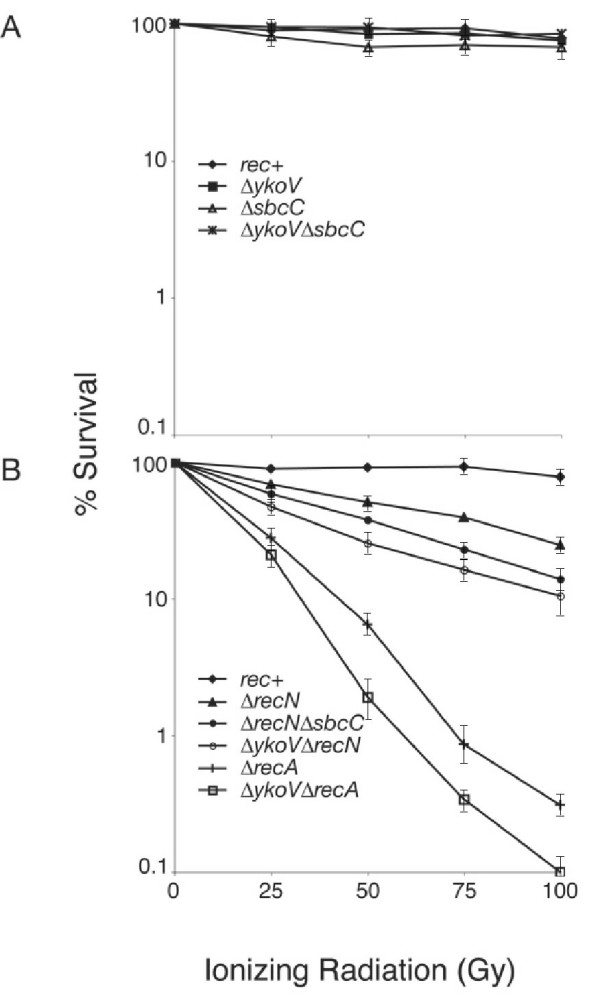
DNA repair sensitivity to γ-radiation of wild type (rec^+^) or of mutant cells grown to stationary-phase. Wild type cells and their isogenic derivatives (Δ*sbcC*, Δ*ykoV*, Δ*sbcC *Δ*ykoV*, Δ*recN*, Δ*sbcC *Δ*recN*, Δ*ykoV *Δ*recN*, Δ*recA*, Δ*ykoV *Δ*recA *or Δ*sbcC *Δ*recA*) were exposed to different doses of γ-radiation. At least three independent experiments were performed, bars denote standard deviations.

BLAST searches showed that the *yirY *gene in the *B. subtilis *genome encodes a typical SbcC protein, with a central CXXC motif (residues 537–540), N- and C-terminal ATPase cassette domains and two long coiled coil domains (Fig. 1). The *yirY *gene product is a 128.7 kDa protein with a pI of 5.3, and is most similar to *E. coli *SbcC, and significantly homologous to eukaryotic Rad50 proteins (C-terminal domain: 30% identity/49% similarity to SbcC, 18% identity/26% similarity to *S. cerevisiae *Rad50). *SbcC *is in a putative operon with and downstream of *addB*, *addA *and *sbcD *genes [*E. coli *SbcC forms a complex with SbcD [24]], and upstream of *yisB*. Therefore, we propose to name the *yirY *gene product SbcC of *B. subtilis*, and the gene *sbcC*. To test the function of SbcC, we constructed a gene disruption of *sbcC*. Because the gene downstream of *sbcC*, *yisB*, performs an important function during growth in *B. subtilis *(our unpublished data), we cloned an internal fragment of *sbcC *into an integration vector that contains a xylose inducible promoter (P_*xyl*_) for transcription of downstream genes. Integration of the vector into the *sbcC *gene resulted in the disruption of the gene, while transcription of *yisB *continued through the P_*xyl *_promoter. Disruption of *sbcC *did not show any effect on normal growth of *B. subtilis *cells, but markedly affected DNA repair. When strain JM42 (*sbcC::cm*) was challenged with 50 or 100 ng/ml of Mitomycin C {MMC forms base adducts and DNA inter-strand cross-links (in a ~4:1 ratio), which can lead to single strand gaps and to DSBs [34-37]}, for 20 min, followed by plating on normal LB plates, the survival rate was strongly reduced compared to wild type cells (Fig. 2, 13.1% or 5.6% survival compared with 50% or 28%, respectively). Microscopic analysis of MMC-treated cells did not show any different morphologies between wild type and mutant strains, like exponentially growing cells, MMC-treated cells were still present as chains of cells, in which individual cells were elongated compared to the non-treated cells. To avoid any complication with the induction of prophages after DNA damage, the *sbcC *deletion was moved into strain YB886 that is cured of all inducible prophages [38]. The *sbcC *deletion strain formed fewer and smaller colonies on 125 ng/ml of MMC than wild type cells (rec^+^) growing at 150 ng/ml (Fig. 3, compare panel "150" with "125" ng/ml). Thus, SbcC is involved in DNA repair. However, cells carrying a deletion of the *recA *gene are much more severely affected in this aspect (the latter cells were unable to survive a challenge with 50 ng/ml, and could not efficiently grow on 3 ng/ml, Fig. 3). To test whether the loss of SbcC affects DSB repair, cells were exposed to γ-irradiation. As shown in Fig. 4A, the *sbcC *deletion strain was marginally, but significantly, impaired in the survival of 50 to 150 Gy (note the log scale-plotting of Fig. 4). However, the true importance of SbcC in DNA repair is masked by the action of other proteins, as will become apparent below.

### Genetic interactions of *sbcC*

To gain further insight into the involvement of SbcC in DNA repair, double mutant strains were constructed in the prophage-free YB886 background, and their survival rates were compared with those of wild type cells, of a *recN *deletion (Δ*recN*) strain, of a *ykoV *(encodes the *B. subtilis *Ku protein) deletion strain or of Δ*recA *cells. The deletion of *sbcC*, *ykoV *or *sbcC *and *ykoV *did not alter the growth rate of the culture compared with wild type cells, similar to *sbcC recN *or *ykoV recN *mutant cells. Contrarily, *recA *mutant cells show a strong reduction in growth and plating efficiency [39]. When exponentially growing wild type or mutant cells were exposed to MMC, the strains could be grouped into three different classes, moderately/very/extremely sensitive to MMC.

Figure 3 shows the concentrations of MMC that affected the plating efficiency of *sbcC*, *recN*, or *recA *mutant cells, respectively. *SbcC *or *ykoV *mutant cells and the double *sbcC ykoV *mutant strain were moderately sensitive to MMC, with a growth defect at 150 ng/ml and a moderate sensitivity in the presence of 125 ng/ml of MMC. In contrast, the growth of the wild type was unaffected by the presence of 150 ng/ml of MMC (Fig. 3), but survival was impaired in the presence of 175 ng/ml (data not shown, [8]). The Δ*recN *strain was sensitive to 40 ng/ml of MMC, and the double Δ*sbcC *Δ*recN *or Δ*ykoV *Δ*r*ec*N *strains were considerably more sensitive than the single Δ*recN *mutant (Fig. 3). The Δ*recN *strain showed confluent growth at the 10^-2 ^dilution (2^nd ^row) and about 50 microcolonies at the 10^-3 ^dilution (3^rd ^row), whereas the double Δ*sbcC *Δ*recN *or Δ*ykoV *Δ*recN *strains neither showed confluent growth at the 10^-2 ^dilution, nor microcolonies at the higher dilution. The higher sensitivity of the double mutant strains can be considered a non-epistatic interaction, suggesting that SbcC acts on a DNA repair pathway different from RecN.

One pathway of DNA repair via HR in *E. coli *involves the RecBCD complex, whose analog in *B. subtilis *is the AddAB complex [40,41]. The survival rate after MMC treatment was reduced in *addAB *mutant cells (29% or 12%, 50 or 100 ng/ml of MMC), compared to wild type cells (Fig. 2). However, *addAB sbcC *mutant cells were more severely affected in the survival test compared with the single mutants (Fig. 2). Therefore, SbcC is also not epistatic with AddAB. We also performed similar experiments using bleomycin that induces DSBs, which yielded similar results (data not shown).

To substantiate these interactions, and to test for sensitivity to DNA damage using another method, stationary phase cultures of wild type or of mutant cells were exposed to ionizing radiation. Wild type, Δ*sbcC*, Δ*ykoV*, or Δ*sbcC *Δ*ykoV *cells exhibited significant resistance to irradiation up to a dose of 100 Gy (Fig. 4A). At higher doses, the lethal dose leaving 50% survivors (LD_50_) was ~147 Gy for wild type cells, ~140 Gy for Δ*ykoV *cells, ~128 Gy for Δ*sbcC *cells, and ~130 Gy for Δ*sbcC *Δ*ykoV *double mutant cells. This finding differs from previous work reporting that the LD_50 _is ~180 Gy for wild type and ~73 Gy for the Δ*ykoV *strain [4]. It is possible and even likely that this discrepancy is due to different strain backgrounds, i.e. the presence of naturally inducible prophages in the strains used in the cited work. Additionally, differing numbers of spores formed by the two different parent strains could account for the discrepancy, because Bs-Ku plays an important role in DNA repair in spores [42].

The Δ*recN *strain was very sensitive to γ-radiation, with a LD_50 _of ~92 Gy. However, the LD_50 _was ~61 Gy for the Δ*sbcC *Δ*recN *strain, and ~40 Gy for the Δ*ykoV *Δ*recN *strain (Fig. 4B), representing a 1.5-fold and 2.3-fold increase in the sensitivity of the double mutant strains compared to the Δ*recN *strain. These results confirm that *scbC *and *recN *or *ykoV *and *recN *show a non-epistatic interaction.

The Δ*recA *strain was highly sensitive to γ-radiation, with a LD_50 _of ~20 Gy (Fig. 4B), however, the LD_50 _of the double Δ*ykoV *Δ*recA *mutant strain was ~16 Gy. The sensitivity of the double mutant strain was clearly higher than that of the already sensitive single mutant and can be considered a non-epistatic interaction, consistent with a previous report [4]. In contrast, the Δ*sbcC *Δ*recA *double mutant strain was as sensitive to gamma irradiation as the *recA *mutant strain (data not shown, see Δ*recA *in Fig. 4).

The data show that SbcC plays an important role in DNA inter-strand cross-link repair, and acts in a different pathway than AddAB or RecN, while it is epistatic with RecA. Its importance becomes clearer in the Δ*recN *background, which is also true for the function of Bs-Ku protein. The data also suggest that HR is the main repair system for DNA inter-strand cross-links and DSBs in *B. subtilis*, while NHEJ constitutes a backup system that is required in the absence of HR repair.

### SbcC forms defined foci on the nucleoids upon induction of DNA DSBs

**Figure 5:**
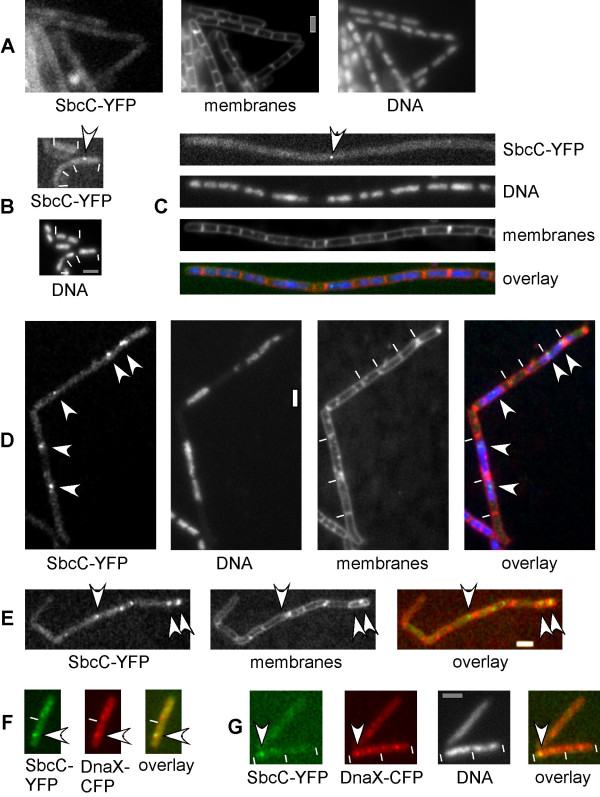
Fluorescence microscopy of *Bacillus subtilis *cells. A-B) Strain JN6 (*sbcC-yfp*) growing exponentially, or C) 30 min or D) 120 min after addition of 50 ng/ml of MMC, or E) 120 min after addition of 200 ng/ml of MMC. F-G) Strain JM61 (*dnaX-cfp*, *sbcC-yfp*) 120 min after addition of MMC. White arrowheads indicate the localization of SbcC-YFP and co-localization of the protein fusions. White lines indicate septa between cells. White bars 2 μm.

To visualize the subcellular localization of *B. subtilis *SbcC, we created a C-terminal fusions of *sbcC *to *gfp *or to *yfp*, ensuring transcription of the downstream genes by an internal xylose-inducible promoter. The SbcC-GFP and SbcC-YFP fusions were fully functional as judged by their ability to survive a challenge with MMC like wild type cells (Fig. 2, and data not shown). 30 min after the addition of MMC, nucleoids adopted a somewhat more condensed morphology and appeared to fuse, because only 4% of MMC treated cells contained two nucleoids, in spite of their increased cell length (compare Fig. 5A with 5C), whereas 21% of exponentially growing cells contain two nucleoids rather than one. 2 hours after induction of DNA damage, cells were highly elongated, and frequently contained nucleoids with abnormal morphology (Fig. 5D and 5E). Exponential growth resumed about 3 hours after addition of 50 to 100 ng/ml of MMC.

In exponentially growing cells, extremely faint fluorescence above background was detectable for SbcC-YFP, which appeared to be present throughout the cells (Fig. 5A). However, in 2% of the cells, discrete SbcC-YFP foci were visible (Fig. 5B). Interestingly, the number of SbcC-YFP foci increased as soon as 30 min after addition of MMC (Fig. 5C, in 5% of the cells, >250 cells analyzed), and was highest 2 hours after addition of MMC (40% of the cells containing foci, >300 cells counted, Fig. 5D). Exposure times of 3 s were necessary to visualize SbcC-YFP foci because signal intensity was very weak (exposure of more than 3 s did not increase signal intensity, as is the case for any GFP fusion analyzed so far with our technical setup). About 90% of the SbcC-YFP foci were located on the nucleoids, showing that the foci were generally associated with DNA. To investigate if SbcC-YFP foci are only induced through inter-strand cross links, or also through DSBs, exponentially growing cells were irradiated with 12 Gy of hard X-rays, which is a dose that induces DSBs but does not reduce viability of *B. subtilis *cells. Similar to the addition of MMC, hard X-rays induced fluorescent foci in 30 to 35% of the cells (data not shown), showing that SbcC foci are also formed after generation of radiation-induced DSBs.

**Figure 6:**
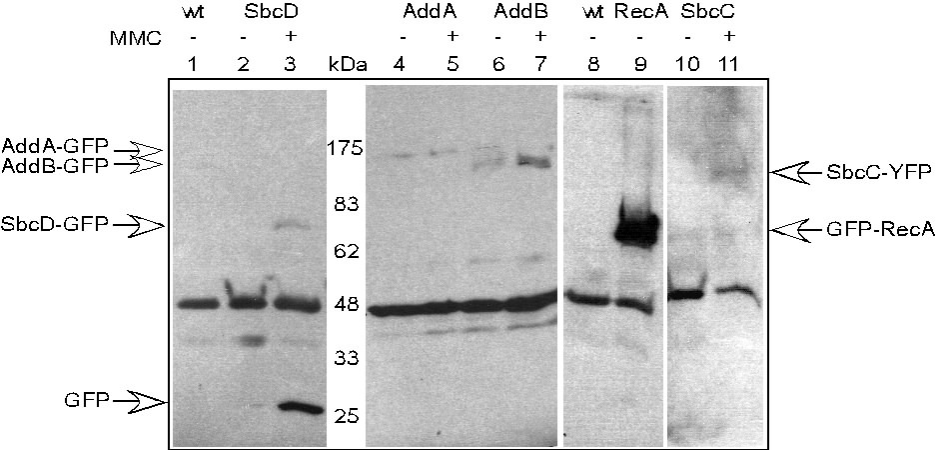
Western blot analysis of *B. subtilis *cells using anti-GFP antiserum. Lane 1: PY79, exponential growth, lanes 2 and 3: JN4 (*sbcD-gfp*), lane 2 exponential growth, lane 3 one hour after addition of MMC (100 ng/ml), lanes 4 and 5: JN7 (*addA-gfp*), lane 4 exponential growth, lane 5 one hour after addition of MMC, lanes 6 and 7: JM41 (*addB-yfp*), lane 6 exponential growth, lane 7 one hour after addition of MMC, lane 8: PY79, lane 9: DK39 (*gfp-recA*), lanes 10 and 11: JN6 (*sbcC-yfp*), lane 10 exponential growth, lane 11 one hour after addition of MMC. The same amount of protein was loaded in each lane. Arrows indicate the position of fusion proteins and of free GFP. AddA-GFP: 169 kDA, AddB-YFP: 162 kDa, SbcC-YFP: 153 kDa, SbcD-GFP: 71 kDa, GFP-RecA: 66 kDa, GFP: 28 kDa. Note that the antiserum strongly cross reacts with a protein of about 48 kDa.

To rule out that the formation of SbcC foci is caused by an artefact, we performed Western Blot analysis of cells growing in the absence or presence of MMC. Fig. 6 shows that the synthesis of full length SbcC-YFP is induced after administration of DNA damage (lane 11), while it is undetectable in cells growing in the absence of damage (lane 10) or in cells lacking the YFP fusion (lane 8), in agreement with the cell biological data. Similar experiments with the same anti GFP serum showed much stronger signals for GFP-RecA (lane 9), or for various other GFP fusions (e.g. BsSMC-GFP or Spo0J-GFP, data not shown) that are not highly expressed in growing cells [43,44], suggesting that SbcC is a poorly expressed protein in growing cells.

After addition of 50 ng/ml of MMC, only one SbcC-YFP focus was detectable in 34% of the cells, while 6% of the cells contained 2 foci, and 60% no visible focus. Interestingly, the number of cells containing foci and the number of foci per cell did not vary significantly between sublethal and lethal doses of MMC (50 to 200 ng/ml, compare Fig. 5D and 5E).

### SbcC centers mostly co-localize with the replication machinery

Inter-strand cross-links block the progression of the replication fork. Also, DSBs can arise at the replication fork, when DNA lesions hinder the progression of the replicative polymerase complex. To test whether SbcC acts at the replication fork, or also at other sites within the cell, we created a dually labelled strain, expressing DnaX-CFP (the tau subunit of DNA polymerase III) and SbcC-YFP. It was technically challenging to obtain clear CFP and YFP signals within the same cell, mostly due to low SbcC-YFP signal intensity. However, we were able to obtain clear signals in both channels for 32 cells (out of 300 cells analyzed). Interestingly, when inter strand cross links were induced, 22 of the SbcC-YFP foci co-localized with DnaX-CFP (Fig. 5F–G), and 4 foci were in close proximity (< 0.3 μm), while 6 of the foci were clearly separate (> 0.3 μm). Thus, SbcC foci mostly co-localize with the replication machinery.

It is interesting to note that the pattern of localization of the replication machinery was altered upon induction of DNA damage. 85% of exponentially growing cells contained one single or two bipolar DnaX-CFP foci (and 15% no signal), whereas after addition of MMC, only 62% of the cells contained this proper localization of the replisome, and 23% of the cells contained signals at various places in the cell (15% did not show any signal, data not shown). Additionally, 66% of exponentially growing cells contained one central DnaX-CFP focus, and 19% two bipolar foci (15% did not contain a signal), whereas after induction of damage, a higher proportion of the cells (40%) contained two or more foci, rather than one (45%, 15% did not contain any focus). Thus, the specific localization of the replication factory becomes more random during DNA repair, and, apparently, replication forks separate (and/or replication reinitiates in the absence of cell division) in a considerable fraction of the cells. Our finding that SbcC forms no more than 1 or 2 foci after induction of DNA damage agrees with the finding that SbcC generally colocalizes with the replication machinery.

### The AddAB complex localizes throughout the cells

**Figure 7:**
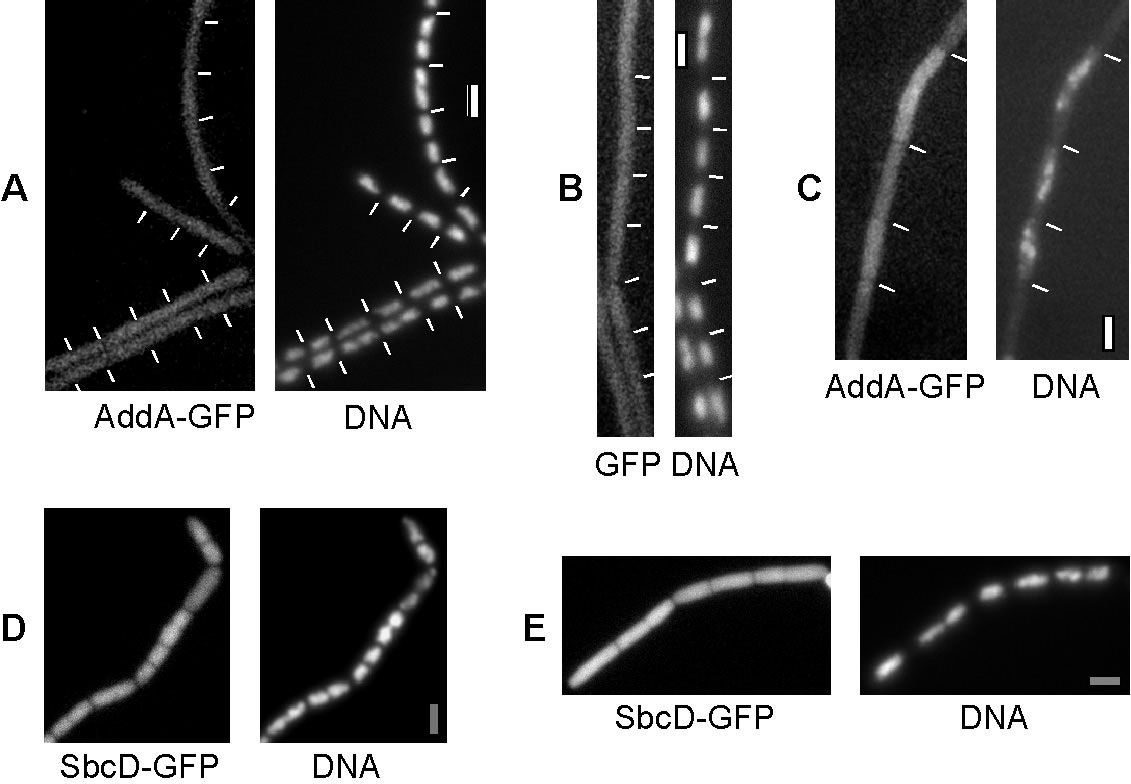
Fluorescence microscopy of *Bacillus subtilis *cells. A-C) Strain JN7 (*addA-gfp*) growing exponentially (A), or B) 30 min or C) 120 min after addition of 50 ng/ml MMC, D-E) strain JN4 (*sbcD-gfp*) growing exponentially (D), or 60 min after addition of MMC (E). White bars 2 μm.

To visualize the other repair proteins from the *add *operon, we generated C-terminal fusions of *addA*, of *addB *or of *sbcD *to GFP, or to YFP, such that *addA*-*gfp*, or *addB*-*yfp*, or *sbcD-gfp *were integrated at the corresponding original locus within the *addAB *operon. AddA-GFP or AddB-YFP fusion proteins were fully functional, as judged by their ability to survive MMC treatment like wild type cells (data not shown). In exponentially growing cells, weak background fluorescence was detectable throughout the cells for AddA-GFP or for AddB-YFP (Fig. 7A, and data not shown). Between 30 min and 2 hours after addition of MMC, fluorescence increased throughout the cells for both fusions, without any apparent accumulation within the cell (Fig. 7B and 7C). Fig. 6 shows that only full-length fusion proteins were produced (lanes 4–7) at a very low level, and at least AddB-YFP clearly accumulated after addition of MMC (compare lane 7 with lane 6). Thus, the AddAB complex does not appear to be specifically associated with the nucleoid, and does not accumulate as discrete foci after induction of DNA damage. Possibly, only few AddAB molecules are recruited to sites of DNA repair. In cells expressing SbcD-GFP, fluorescence was detected throughout the cells, and increased in level after addition of MMC (Fig. 7D and 7E), however, much more free GFP than SbcD-GFP fusion protein was apparent in the cells (Fig. 6, lane 3). These results show that the SbcD-GFP fusion is proteolytically cleaved (the first such event we have found so far), such that freely diffusing GFP is masking the localization of SbcD-GFP. Like SbcC-YFP, the signal of SbcD-GFP/GFP increased after addition of MMC (Fig. 6, compare lane 3 with lane 2).

## Discussion

Our work shows that the bacterial counterpart of the eukaryotic Rad50 protein, SbcC, confers an important function in the repair of inter strand cross links in DNA caused by MMC, and plays a role in the repair of DSBs (caused by gamma irradiation), most likely in the context of repair of collapsed replication forks. The deletion of *sbcC *led to a considerable sensitivity to DNA damage. However, the loss of SbcC in a *recN *or in an *addAB *mutant background increased the sensitivity to DNA damage of the very sensitive *recN *or *addAB *single mutant strains. Contrarily, the loss of SbcC in *recA *mutant cells did not increase the extreme sensitivity of the single *recA *mutant strain, showing that SbcC is epistatic with RecA, but not with RecN or with AddAB. These experiments suggest that SbcC acts on DNA repair via homologous recombination (HR), and show that it confers a function distinct from RecN or from AddAB.

Interestingly, like SbcC, Bs-Ku protein was non-epistatic with RecN or AddAB, and the loss of BS-Ku in *recN *mutant cells led to a similar increase in sensitivity towards DNA damage than the loss of SbcC in this mutant background. Because the loss of Bs-Ku protein in a *sbcC *mutant strain did not increase the mild sensitivity of the *sbcC *single mutant strain, and vice versa, it is possible that SbcC may also play a role in NHEJ. Possibly, upon encountering DNA ends, the SbcC alone, or in concert with the Bs-Ku protein, might tether DNA ends together, which are finally joint by a DNA ligase [45]. Indeed, several reports have supported a dual function of the eukaryotic Rad50 complex in DNA repair via HR as well as in NHEJ. However, many more experiments are required to establish a connection between SbcC and NHEJ, and the finding that Bs-Ku is non epistatic with RecA, in contrast to SbcC, shows that the main function of SbcC does not lie in NHEJ, but rather in HR.

It is clear from our work that repair of DSBs and of inter-strand cross-links via HR plays a major role in *B. subtilis *cells, because the loss of proteins participating in HR [e.g. RecN, AddAB] caused severe sensitivity to radiation-induced DNA damage, while the loss of Bs-Ku led to moderate sensitivity. The activity of Bs-Ku became important in cells defective in repair via HR, indicating that NHEJ serves as a back up system to repair DSBs that can not be fixed via HR, e.g. non-duplicated chromosome regions, for which no sister chromosome is present to set up HR. Interestingly, it has recently been shown that Bs-Ku and DNA ligase encoded by *ykoU *play an important role in resistance of spores to killing through dry heat, a treatment that introduces breaks into DNA [42]. Because the spore contains only a single chromosome, DSBs can not be repaired via HR, but only through NHEJ, which is also important for DSB repair during cell cycle exit in all other types of cells. However, our results show that Bs-Ku also performs a function in DNA repair in exponentially growing cells, because the absence of Bs-Ku leads to mild but noticeable sensitivity towards MMC.

A further major finding of this work that provides a clue as to the function of SbcC is the observation that SbcC transiently assembles into discrete subcellular centers on the nucleoids after the induction of inter-strand cross-links and of DSBs. In growing cells, SbcC was mostly dispersed throughout the cells, while the accumulation of SbcC was observed as early as 30 min after induction of DNA damage, its main accumulation occurring between 1 and 2 hours post induction. Thereafter, the SbcC-GFP foci dissipated, concomitant with the resumption of growth. Intriguingly, SbcC assembled most frequently at the replication machinery, suggesting that SbcC acts on DNA damage occurring at the replication forks, which can block replication (e.g. cross links or base adducts), or lead to fork collapse (e.g. when forks run into ssDNA gaps) [46]. However, at low frequency, replication forks collapse in non-stressed exponentially growing cells [47]. Consistent with this, we also observed SbcC foci at low frequency in exponentially growing cells in the absence of externally induced damage. The *E. coli *SbcCD complex, which shows some similar *in vitro *activities than the Rad50 complex [48], has been shown to be involved in the cleavage of DNA hairpins that can occur at the replication forks [24]. Possibly, this is one of the specific roles SbcC confers during replication, and at stalled or collapsed replication forks, SbcC could be involved in replication restart through HR, and/or removal of inter-strand cross-links.

Recently, we have shown that RecN assembles at defined DSBs or at random DNA lesions as early as 15 min, while RecO and RecA are recruited 30–60 min after induction of DSBs [31,32]. DSB repair appears to last for about 3 hours, and RecF protein is recruited to DSBs after 90 min, thus representing a late-recruited protein. RecN binds to ssDNA at an internal site, and gathers the 3'-hydroxyl ssDNA ends *in vitro *[8]. These finding are consistent with the idea that RecN, like eukaryotic Rad50 [49], might be an early sensor of sites of DNA damage on replicating and non-replicating chromosomes [50]. The cytological data presented here provide a clear distinction between the functions of SbcC and of RecN, because RecN is generally not associated with the replication machinery [32], and appears to act as a nucleation factor of DNA repair centers away from the replication machinery, while SbcC appears to assemble at DNA damage occurring at the replication factory. A different function for SbcC and RecN – dependent on whether DNA damage occurs at the replication fork or elsewhere on the nucleoid – can explain why SbcC-YFP foci are only observed in about 40% of the cells after induction of random DNA damage (this work), while RecN-YFP foci occur in about 70% of the cells (but not in all cells) under these conditions [31].

SbcC and RecN belong to the SMC protein family, whose members are key players in various chromosome dynamics. Our report establishes that these two *B. subtilis *proteins SMC like proteins confer important yet distinct functions in the repair of DNA base modifications, of DNA inter-strand cross-links and of DSBs. It will be interesting to elucidate the molecular basis of their different modes of action.

## Methods

### Bacterial strains and media

*E. coli *XL1-Blue (Stratagene) harbouring plasmids were grown in Luria-Bertani (LB) rich medium supplemented with 50 μg/ml ampicillin. *B. subtilis *strains were grown in LB rich medium, and when necessary with appropriate antibiotics. For microscopy, cells were grown in S7_50 _defined medium [51]. All *B. subtilis *strains used in this study are listed in Table 1.

### Construction of plasmids and strains

All CFP, GFP or YFP fusions were done by PCR amplification of the 3' regions of each gene, and cloning into a vector containing a C-terminal *cfp*, *gfp *or *yfp *gene. All fusion vectors contained inducible promoters ensuring transcription of downstream genes. To create a C-terminal fusions of AddA and of SbcD to GFP, the corresponding 3' regions (about 500 bp) were PCR amplified (AddA-GFP 5'-ATTCGGGCCCAGCTGAGCTGGACCTAC-3' and 5'-TCGGAATTCACCACCGCCT-AATGTCAGAATGTGCCC-3', SbcD-GFP 5'-TCGGAATTCACCGCCTTTCGCA-TCCTCCTCTTCAAC-3' and 5'-ATTCGGGCCCTTTGCATCGCCCGCAAACG-3') and were cloned into *Apa*I and *Eco*RI sites of pSG1164 [52], giving rise to plasmids pA1164 and pD1164, respectively. The 3' region of *yirY *(*sbcC*) (primers 5'-ATTCAAGCTTGCAAA-CTTGAAAACGAG-3' and 5'- TCGGAATTCACCACCGCCCATCAACTCAAGTGATAC-CCG- 3') was cloned into pSG1187 (C-terminal *yfp*) using *Hind*III and *Eco*RI sites, giving rise to plasmid pC87. This plasmid could not be integrated into the *B. subtilis *chromosome because of the downstream *yisB *gene. An *Eco*RI *Spe*I fragment from pC87 containing the 3'*yirY-yfp *fusion was cloned into pSG1164, giving rise to pC87xyl. The 3' region of *addB *(primers 5'-CCATCGATACCGCCTCCGGAATGTTCATTGCCATC -3' and 5'-TTGATTTATCGATTACACATTC -3') was cloned into pMutinYFP (containing an IPTG inducible promoter for downstream genes) using *Cla*I, giving rise to plasmid pBy. All plasmids were transformed into wild type (PY79) *B. subtilis *cells selecting for chloramphenicol (Cm) resistance (5 μg/ml). All fluorescent tag vectors were integrated into the *B. subtilis *chromosome via single crossover integration, which was verified by PCR and Western Blot analysis.

For disruption of *sbcC*, a 1000 bp internal fragment of *sbcC *was PCR amplified (primers 5'-ACATGCATGCAATCTGCCCTTCGCCTCTTG-3' and 5'-TCATAAGCTT-CAAACAGGAACAGCTTTCACG-3') and was cloned into *Hind*III and *Sph*I sites of pJQ43 (containing a constitutive promoter for downstream genes) [53], giving rise to plasmid pSbcKO. Single crossover integration of pSbcKO lead to disruption of the *sbcC *gene (*sbcC::cm*) and to the synthesis of a C-terminal truncated SbcC. For the disruption of *ykoV*, a 480 bp internal fragment from *ykoV *was cloned into pSG1164, and the resulting plasmid pykoVKO was transformed into PY79, generating strain JH1 (*ykoV::cm*).

The *addAB sbcC *triple mutant strain was generated by transforming *addA5 addB72 *(collectively termed *addAB *mutant cells) mutant cells [54] with chromosomal DNA from strain JM42, selecting for Cm resistance. The inactivation of the *recN *and *recA *genes by a double crossover event was previously described [41,55]. To study genetic interactions in a strain freed of inducible prophages (which complicate analysis of DSBs), all deletions were moved into strain YB886 (*rec*^+ ^control) [38]. The Δ*sbcC *and Δ*ykoV *strains were generated by transferring the *sbcC::cm *or *ykoV::cm *allele onto the YB886 strain by a single crossover event. To generate double mutants, the Cm resistance cassette in Δ*sbcC *or Δ*recN *cells was replaced by tetracycline resistance *in vivo *by transformation with plasmid pCm::tet (BGSC, Wisconsin, USA). Double mutants were produced by transformation of Δ*recN*, Δ*recA*, Δ*sbcC *and Δ*ykoV *mutant strains with the appropriate chromosomal DNA from other mutant strains.

### DNA repair sensitivity studies

DNA modifications generated by Mitomycin C (MMC) result in the formation of base adducts and inter-strand cross-links, which generate single-strand breaks and DSBs [35-37]. Ionizing radiation (γ-rays) leads to the formation of apurinic/apyrimidinic sites and generates nicks and DSB. All survival studies were performed at least 3 times, and were performed in the absence of antibiotics. For sensitivity to MMC assays (Fig. 3), exponentially growing *B. subtilis *cells were obtained by inoculating overnight cultures in fresh LB media and by growing to an A_560nm _of 0.4 at 37°C. Appropriate dilutions (10 μl of serial 10-fold dilutions, 1 × 10^-2 ^to 1 × 10^-5^) were spotted onto LB plates supplemented with the indicated concentration of MMC, and were incubated overnight at 37°C. MMC and all plates used for survival tests were kept in the dark at all times, in order to avoid complications generated by photoadducts.

For ionizing radiation assays (Fig. 4), stationary phase cultures of *B. subtilis *cells were obtained by inoculating overnight cultures in fresh LB media and by growing to an A_560nm _of 2 at 37°C. Irradiation was carried out with ^137^Cs γ-rays using a Mark I irradiator (JL Shepard & Associates) at a dose-rate of 3.7 Gy/min, without shaking at room temperature, using 50 μl of cells from a 100-fold dilution. Appropriate dilutions of irradiated cells were plated, incubated overnight at 37°C and colonies were counted from at least three independent experiments.

### Western blot analysis

Western blotting was performed using same amounts of cell extracts, as determined by Bradford tests, using antiserum generated against purified GFP-6-his protein (kind gift from David Rudner, Harvard Medical School).

### Image acquisition

Fluorescence microscopy was performed on an Olympus AX70 microscope. Cells were mounted on agarose pads containing S750 growth medium on object slides. Images were acquired with a digital MircoMax CCD camera; signal intensities and cell length were measured using the Metamorph 4.6 program. DNA was stained with 4',6-diamidino-2-phenylindole (DAPI; final concentration 0.2 ng/ml) and membranes were stained with FM4-64 (Molecular Probes, USA, final concentration 1 nM).

## Authors' contributions

JM constructed strains and performed fluorescence microscopy, HS constructed strains and carried out sensitivity assays, ST and DK carried out sensitivity assays, MK performed Western blot analyses, JCA and PLG conceived of the study, and participated in its design and coordination. All authors read and approved the final manuscript

## Figures and Tables

**Table 1:** Strains used in this work

Strain	genotype	reference
PY79		[58]
JN4	*sbcD-gfp*	this work
JN6	*sbcC-yfp*	this work
JN7	*addA-gfp*	this work
JM41	*addB-yfp*	this work
JM42	*sbcC::cm*	this work
JH1	*ykoV::cm*	this work
JM61	*dnaX-cfp*, *sbcC-yfp*	this work
YB886	*trpC*2 *metB*5 *amyE sigB*37 *xin*-1 *att*SPβ	[38]
BG189	*addA5 addB72*	[54]
BG277	*recN::cm*	[59]
BG190	*recA::cm*	[55]
BG809	*ykoV::cm*	this work
BG811	*sbcC::cm*	this work
BG843	*ykoV::tet*, *recN::cm*	this work
BG849	*ykoV::tet*, *recA::cm*	this work
BG847	*ykoV::cm*, *sbcC::tet*	this work
BG845	*sbcC::tet*, *recN::cm*	this work
DK39	*gfp-recA*	[32]

Note that BG strains are isogenic with the YB886 wild type strain (used for survival studies in Fig. 2 and 3), while the other strains are isogenic with the PY79 wild type strain (used for Fig. 1 and localization studies).
